# Cryptographically Secure Pseudo-Random Number Generator IP-Core Based on SHA2 Algorithm †

**DOI:** 10.3390/s20071869

**Published:** 2020-03-27

**Authors:** Luca Baldanzi, Luca Crocetti, Francesco Falaschi, Matteo Bertolucci, Jacopo Belli, Luca Fanucci, Sergio Saponara

**Affiliations:** Department of Information Engineering, University of Pisa, Via G. Caruso n. 16, 56122 Pisa, Italyluca.fanucci@unipi.it (L.F.); sergio.saponara@unipi.it (S.S.)

**Keywords:** intelligent sensors, autonomous driving, cyber security, HW accelerator, on-chip random number generator (RNG), SHA2, FPGA, ASIC standard-cell

## Abstract

In the context of growing the adoption of advanced sensors and systems for active vehicle safety and driver assistance, an increasingly important issue is the security of the information exchanged between the different sub-systems of the vehicle. Random number generation is crucial in modern encryption and security applications as it is a critical task from the point of view of the robustness of the security chain. Random numbers are in fact used to generate the encryption keys to be used for ciphers. Consequently, any weakness in the key generation process can potentially leak information that can be used to breach even the strongest cipher. This paper presents the architecture of a high performance Random Number Generator (RNG) IP-core, in particular a Cryptographically Secure Pseudo-Random Number Generator (CSPRNG) IP-core, a digital hardware accelerator for random numbers generation which can be employed for cryptographically secure applications. The specifications used to develop the proposed project were derived from dedicated literature and standards. Subsequently, specific architecture optimizations were studied to achieve better timing performance and very high throughput values. The IP-core has been validated thanks to the official NIST Statistical Test Suite, in order to evaluate the degree of randomness of the numbers generated in output. Finally the CSPRNG IP-core has been characterized on relevant Field Programmable Gate Array (FPGA) and ASIC standard-cell technologies.

## 1. Introduction

The rapid technology development of intelligent sensors in the automotive field in recent years, driven by institutions and supported by manufacturers to integrate advanced systems for active safety and hazard prevention, has generated additional collateral technological needs. All the equipment distributed on board the vehicle are, in fact, interconnected with real communication networks and exchange critical data and sensitive information that must be protected from potential attacks and violations. Cybersecurity thus becomes a central issue and all mechanisms ensuring authentication, confidentiality and integrity of messages become an enabling technology for the further development of Advanced Driver Assistance Systems (ADAS) and Autonomous Driving Systems (AD).

In particular, projects like the one carried on in the framework of the European Processor Initiative (EPI) programme [1] anticipate requirements from future scenarios, with very high throughput hardware accelerators for cybersecurity applications. Highly automated vehicles will be equipped with advanced sensors (e.g., camera arrays, radar, lidar), capable of generating significant data flows. Moreover, over-the-air update (OTA) of the software of control units will be implemented, for which it will be very important to keep the update time as short as possible. In this context, it is essential to increase information security, and it is therefore necessary to implement encrypted data transfers (i.e., encryption and decryption) and verified content (i.e., digital signature) with throughput compatible with the use of buses such as automotive ethernet (i.e., 1–10 Gbps). For this reason, hardware accelerators with throughput requirements of the order of tens Gbps are necessary, also to take into considerations the expected lifespan of vehicles.

Random numbers are widely used in encryption and security applications, usually to generate encryption keys or secret data to be shared between communication entities. Therefore a Random Number Generator (RNG) is a very important primitive for cryptographically secure applications [2]. In particular, it is used as a fundamental part for the development of several globally distributed applications related to the field of cyber security such as digital payments, online authentication and instant messaging [3]. Cryptographic keys are based on random numbers and must be characterized by a high degree of unpredictability to be considered secure: this is necessary to prevent an attacker from violating the security chain based on this cryptographic key. Random numbers are also the starting point for generating nonces within authentication protocols as a countermeasure against replay attacks, so the higher the degree of randomness, the more robust the countermeasure will be. Moreover, strong random number generation helps digital signature procedures to prevent private keys to be disclosed, thus violating the signature itself [4]. Several development techniques for RNG engines are reported in literature, and many of them exploit physical sources (e.g., analog noise) as random processes to obtain the randomness characteristic of the generated bit sequences [3,4,5,6,7,8,9].

These circuits are identified as True Random Number Generators (TRNG) and their output sequences are considered high quality random numbers. However, TRNGs have non-negligible disadvantages that must be considered: the use of physical sources leads to high energy consumption and insufficient throughput for fast and advanced integrated systems. TRNGs are also sensitive to changing operating conditions, which means that post-processing must be implemented to ensure reliable random output data, further reducing the throughput under non-ideal condition.

To overcome these limitations, a powerful Deterministic Random Bit Generator (DRBG) circuit can be used in addition to a very low-area, low-power and low-throughput TRNG implementation. This means that the RNG engine would be mainly based on a deterministic algorithm that generates pseudo-random output sequences. In this case, the required degree of randomness is obtained through additional mechanisms to increase the level of entropy of the generated sequences, which would otherwise be deterministic. This operation is called *reseed* and it consists in providing a trigger to restart the circuit of the deterministic algorithm from a new high entropy starting point (i.e., the new seed). DRBG-based solutions use periodic *reseed* to allow the RNG to generate pseudo-random binary output sequences that are equivalent and indistinguishable from true random ones. The limitations of TRNGs for high-speed devices are thus overcome by restricting its use to the periodic seed generation operation only, which has the characteristic of being a very light task. For this reason the new seed is usually provided by a very low complexity and target specific TRNG module [10], which also may be powered down when not necessary.

For the proposed architecture, the DRBG mechanism was chosen from those approved by National Institute of Standards and Technology (NIST) [6]. The standard provides general information for PRNGs based on cryptographic primitives, some of which are incontrovertible and proven (e.g., Hash DRBG and HMAC DRBG). For the proposed Cryptographically Secure Pseudo-Random Number Generator (CSPRNG) IP-core the algorithm selection was made based on a compromise between performance, area and security strength. The Hash DRBG with SHA-256 as cryptographic core (i.e., based on the SHA2 algorithm) proved to be the most efficient solution between logical complexity and expected throughput during random bit generation, offering 256 bits of security strength.

The reminder of this paper is organized as it follows: Section 2 presents the trade-off analysis among the different suitable DRBG algorithms, Section 3 details the implementation of the SHA2 algorithm being chosen to develop the DRBG core, Section 4 describes the Hash DRBG design architecture as CSPRNG IP-core, Section 5 collects the characterization results. Finally, conclusions are discussed in Section 6.

## 2. DRBG Algorithms Trade-Off Analysis

As mentioned in Section 1, the different deterministic algorithms suitable for implementing DRBG circuits have been evaluated by the NIST and those approved-recommended are collected in the NIST SP 800-90A Rev.1 pubblication [6]. Such mechanisms present common features and functionalities:

- they rely on a one-way cryptographic function, thus providing backtracking resistance;
- the internal status memories are secret and inaccessible to the user;
- the following essential operations are allowed:
  - *instance*, to acquire a random seed (i.e., concatenation of input entropy content, possibly input or internal random nonce, and personalization string) and to initialize the internal state to a random value derived from the seed;
  - *reseed*, to acquire a random seed (i.e., concatenation of internal state, input entropy content, and personalization string) and update the internal state to a random value derived from the seed;
  - *generate*, to generate an output bits sequence based on current state and then to update the state to a random value derived from previous state;
  - *uninstantiate*, to delete the internal state;
- they support a maximum security strength (112, 128, 192 or 256) and all of the lower ones;
- a *reseed counter* counter, and the corresponding threshold called *seed lifetime*, is present to signal the user that the mechanism needs a new *seed*;
- user is always able to run a command with an associated personalization string, which needs not to be secret but it contributes to the internal state randomization.

Most of the DRBG engines implementations rely on hash functions and counter mode (CTR) of symmetric-key encryption processes. Hash functions family includes SHA1 and SHA2 algorithms, but the former is going to be deprecated because of its low security strength and high vulnerability [11], therefore only SHA2 cryptographic primitives are taken into exam for Hash DRBG mechanisms. The main parameters related to DRBG cores based on SHA2 primitive are reported in Table 1.

CTR (CTR is abbreviation for *Counter*) DRBG mechanisms are based onto block cipher cores used in *counter mode*. Different block cipher cores are suitable to develop a DRBG circuit and the main parameters related to the different implementations are collected in Table 2.

Table 3 summarizes area and latency values obtained for our version of SHA2 IP-core, where complexity values are related to the characterization on 45nm ASIC standard-cell technology. In the perspective to implement a Hash DRBG circuit, solutions for SHA-224 and SHA-384 are discarded in favor of SHA-256 and SHA-512 because area and latency values are the same, but the former couple offers a shorter output block. Concerning SHA-256 and SHA-512 comparison, the following considerations can be done in order to select the best candidate for Hash DRBG:

- SHA-256 has lower latency per block than SHA-512;
- SHA-512 offers a higher throughput with respect to SHA-256, since it provides 512 bits every 83 clock cycles instead of 256 bits every 67 clock cycles;
- SHA-256 is more compact in terms of area, which reflects also on internal state registers area footprint. As shown in Table 1, seedlen is 440 for SHA-256 and 888 for SHA-512, meaning that the internal state requires around 900 registers for the former and 1800 for the latter.

The throughput values of the SHA-256 core and SHA-512 core, when operating in generation phase for a Hash DRBG implementation, can be obtained through Equations (1) and (2), respectively.

(1) $$T_{SHA-256}=256/67\cdot f_{clk}\cdot n_{parallel\_core}=3.82\cdot f_{clk}\cdot n_{parallel\_core}\;bit/s$$

(2) $$T_{SHA-512}=512/83\cdot f_{clk}\cdot n_{parallel\_core}=6.17\cdot f_{clk}\cdot n_{parallel\_core}\;bit/s$$

Concerning the CTR DRBG circuit, the AES IP-core is proved to be best in class for both area and throughput. Table 4 collects the area and latency values for our versions of AES-128 and AES-256 IP-cores characterized on 45nm ASIC standard-cell technology.

Given that the target is to identify the most suitable core for implementation of DRBG circuit with highest level of security strength possible, the AES-256 algorithm is the only block cipher core being considered for the trade-off. As shown in Table 4, its area is lower than the one for SHA-256, while the throughput value is higher than that reported for SHA-512. In particular, the throughput of AES-256 to be considered for a CTR DRBG implementation is calculated as below:

(3) $$T_{AES-256}=128/15\cdot f_{clk}\cdot n_{parallel\_core}=8.53\cdot f_{clk}\cdot n_{parallel\_core}\;bit/s$$

**Figure 1:**
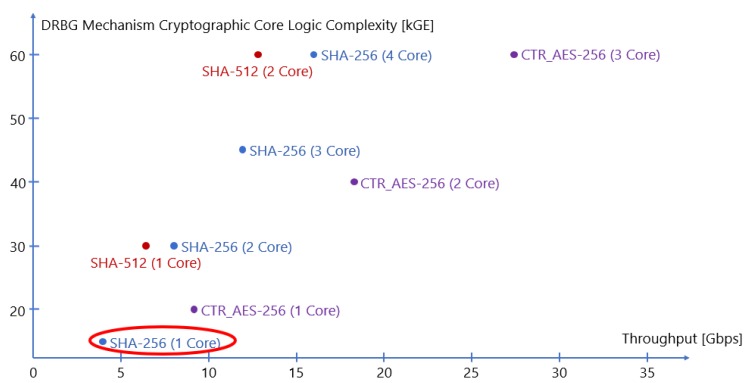
Comparison between NIST approved DRBG mechanisms based on logic complexity in kGE and throughput.

Figure 1 shows a comparison of DRBG mechanisms implemented as in [5] and based on Hash algorithms (i.e., SHA-256 or SHA-512) and block ciphers (i.e., AES-256) used in CTR mode. The logic throughput and complexity values are obtained with synthesis on 45 nm ASIC standard-cell technology.

The algorithm chosen for the development of the DRBG circuit inside the CSPRNG IP-core was SHA-256 (i.e., the primitive SHA2), therefore we decided to use an Hash DRBG despite the better area and latency values collected for AES cores (i.e., for CTR DRBG circuit). This is because M.Schmid [12] explained how block cipher-based DRBGs should not be used as they are indeed not able to reach maximum security strength. The author declares that the pseudo-random permutation inside each AES round, coupled with counter mode of operation, generates a binary sequence which results to be distinguishable with respect to what a random source could give, thus being unable to satisfy the security requirements. This is not the case with Hash-based DBRGs, so the use of SHA-256 cores offers better robustness to the entire security chain where the CSPRNG is based on this algorithm. The SHA-256 core ensures a compact implementation for the mechanism and the possibility to extend the design for supporting multiple cores to increase the throughput. In a context with multiple cryptographic cores, 2 SHA-256 perform better than a single SHA-512, having a higher throughput and requiring lower internal state.

## 3. SHA-256 Core Implementation

As explained in Section 2, the fundamental element of the proposed Hash DRBG circuit is the SHA-256 core, based on SHA2 cryptographic primitive. In order to achieve high throughput for the whole CSPRNG IP-core, it is then essential to optimize the SHA-256 implementation performances to the maximum. To do so, the canonical logic implementation derived from the standard [13] has been improved through the use of *Carry-Save Adder* (CSA) units for consecutive additions and by application of retiming-pipelining to perform delay balancing. To better understand the implemented optimizations, a brief description of the standard is given.

The SHA-256 standard may be defined by two separate, ideally consecutive, procedures:

1. the *message schedule*;
2. the *compression function*.

The *message schedule* is in charge of creating a *key* schedule starting from the 512-bits *input message* to be then provided to the *compression function*. The operation is performed through the $\sigma_{0}$, $\sigma_{1}$ and modulo $2^{32}$ adder operations defined in Equations (4)–(6) respectively.

(4) $$\sigma_{0}\left(x\right)=RotateRight_{7}\left(x\right)⊕RotateRight_{18}\left(x\right)⊕ShiftRight_{3}\left(x\right)$$

(5) $$\sigma_{1}\left(x\right)=RotateRight_{17}\left(x\right)⊕RotateRight_{19}\left(x\right)⊕ShiftRight_{10}\left(x\right)$$

(6) $$x⊞y=x+y\;(\mathrm{mod}\;2^{32})$$

**Figure 2:**
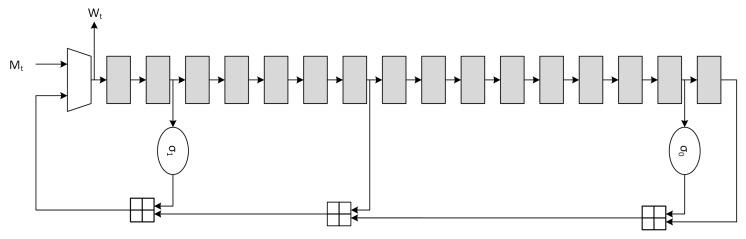
SHA2 standard *message schedule* architecture.

Usually the message schedule operation is also called *expansion* due to the fact that the 512-bits input message is expanded to 32·64=2048bits. The canonical serial architecture of the *message schedule* block, derived from Equation (7), is depicted in Figure 2.

(7) $$W_{t}=\left\{\begin{array}{cc} M_{t}\;\; & 0<t<15\\ \sigma_{1}\left(W_{t-2}\right)⊞W_{t-7}⊞\sigma_{0}\left(W_{t-15}\right)⊞W_{t-16}\;\; & 16<t<63 \end{array}\right.$$

The optimization for the message schedule is performed on the adder chain through the use of CSA, which are essentially *Full-Adder Arrays* (FAA), producing partial sums (*ps*) and shift-carries (*sc*).

(8) $$\begin{array}{cc} \; & ps_{i}=a_{i}⊕b_{i}⊕b_{i}\\ \; & sc_{i}=\left(a_{i}\wedge b_{i}\right)\vee \left(a_{i}\wedge c_{i}\right)\vee \left(b_{i}\wedge c_{i}\right) \end{array}$$

**Figure 3:**
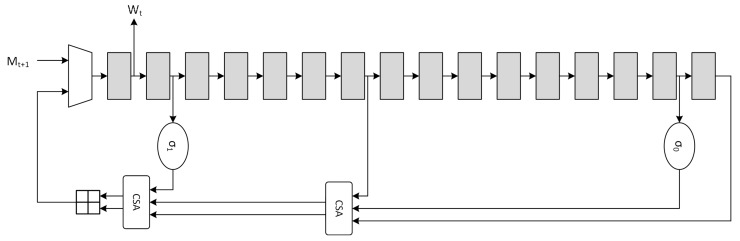
SHA2 optimized *message schedule* architecture.

CSAs have advantages on both area and critical path. Implementation on 45nm and 7nm ASIC standard-cell technologies demonstrated that, when compared to *Carry-Lookahead Adder* (CLA) units, the delay relationship is $T_{CLA-32}=1.78\cdot T_{CSA-32}$ and $T_{CLA-32}=1.87\cdot T_{CSA-32}$ respectively for the two technologies. The optimized serial implementation is shown in Figure 3, where the high-level timing block analysis shows that the critical path is reduced from $T_{\sigma_{0}}+3\cdot T_{⊞}$ to $T_{\sigma_{0}}+2\cdot T_{CSA}+T_{⊞}$. Further optimizations are possible through the use of retiming, but they are not considered due to the critical path being mainly located in the *compression function* architecture.

The SHA-256 *compression function* is composed of three consecutive steps: initialization, one-way compression and termination. In the first step, the variables A-H are initialized with the intermediate Hash value $H^{\left(t-1\right)}$ (the first 512-bits message block at t=1 uses a constant $H^{\left(0\right)}$ provided by the standard). The one-way compression then performs 64 loops according to:

$T_{1}$ $\leftarrow H⊞\Sigma_{1}\left(E\right)⊞Ch\left(E,F,G\right)⊞K_{j}⊞W_{j}$
$T_{2}$ $\leftarrow \Sigma_{0}\left(A\right)⊞Maj\left(A,B,C\right)$
*H* ←G
*F* ←E
*E* $\leftarrow D⊞T_{1}$
*D* ←C
*C* ←B
*B* ←A
*A* $\leftarrow T_{1}⊞T_{2}$

Finally the intermediate Hash value at time *t* is calculated by a $2^{32}$ modulo addition between the variables A-H at initialization time and the variables A-H after the one way compression. The functions Maj, Ch, $\Sigma_{0}$ and $\Sigma_{1}$ are defined as:

(9) $$\begin{array}{cc} \; & Maj(x,y,z)=(x\wedge y)⊕(x\wedge z)⊕(y\wedge z)\\ \; & Ch(x,y,z)=(x\wedge y)⊕(\neg x\wedge z)\\ \; & \Sigma_{0}\left(x\right)=RotateRight_{2}\left(x\right)⊕RotateRight_{13}\left(x\right)⊕RotateRight_{22}\left(x\right)\\ \; & \Sigma_{1}\left(x\right)=RotateRight_{6}\left(x\right)⊕RotateRight_{11}\left(x\right)⊕RotateRight_{25}\left(x\right) \end{array}$$

**Figure 4:**
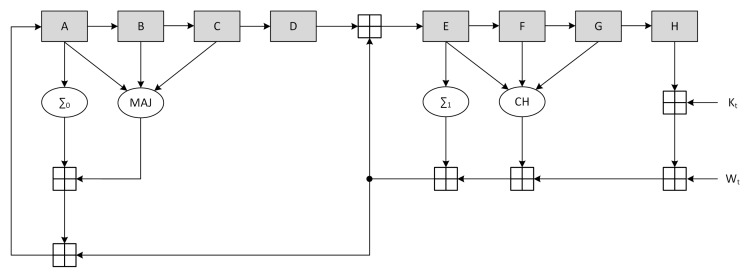
SHA2 standard *compression function* architecture

The canonical scheme corresponding to the described procedure is represented in Figure 4, where the output stage performing the termination phase is not represented.

The high-level block timing analysis showed that the critical path on the non-optimized architecture is located between register H and register E, involving 5 ⊞ operations. Optimization of the *compression function* was achieved through the use of CSA, retiming and delay balancing. In particular, all the adder chains were converted to CSA with the exception of register B and E inputs. Moreover, the path going from $K_{t}$-$W_{t}$ was duplicated to allow the value of register D to be added immediately to $H+K_{t}+W_{t}$. Finally a pipeline stage $L_{1},L_{2}$ was added (with the associated C-D multiplexer to ensure the functionality) and the a register was split to move the CLA position.

Finally, both SHA-2 implementations have been synthesized on 45 nm and 7 nm ASIC technologies, whose results are represented on Table 5 for canonical and optimized architectures.

**Figure 5:**
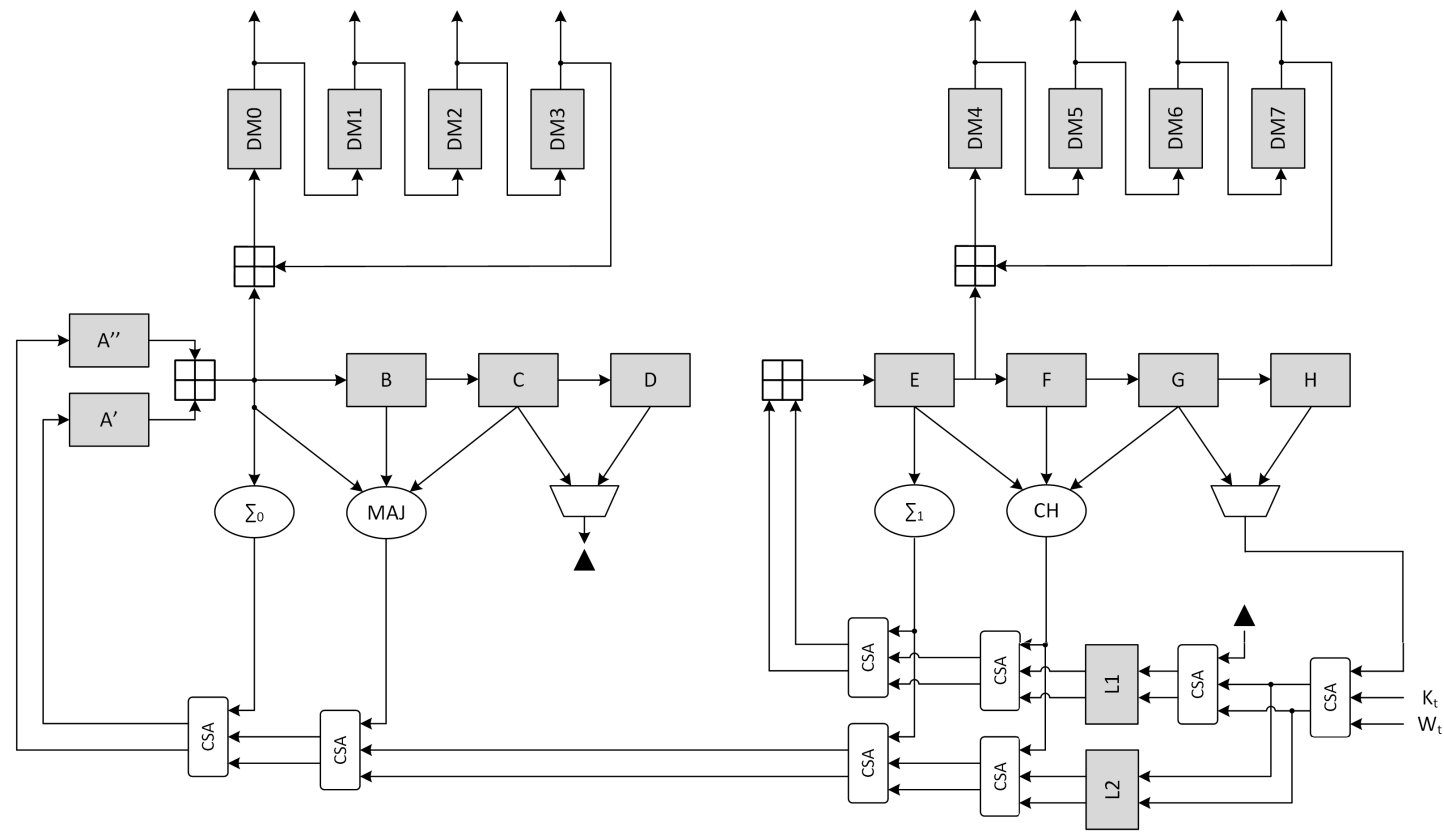
SHA2 optimized *compression function* architecture.

A detail analysis of the critical path on the implemented design shows that the real critical path on 45 nm ASIC technology is located between register L1 and register E (Figure 5), while for the 7 nm ASIC technology it is located in the message schedule between the second right register and the left one (Figure 3). This behavior can easily be attributed to the synthesizer, which is able to use complex ASIC cells and better merge the 4·CSAs than the 2·SCA+1·CLA. a separate synthesis, to emulate the main paths of the canonical and optimized architectures on the 45 nm technology, shows the critical path of these extracted sub-elements. As visible from Table 6 the 2·SCA+1·CLA sub-clock is the slowest path w.r.t. The optimized implementation, with an equivalent frequency $f_{2\cdot CSA+1\cdot CLA}=1200$ MHz.

Looking for a comparison between the canonical ASIC implementation and the ASIC optimized one, the latter results to be about 9% smaller, while providing about a 56% maximum frequency increase.

## 4. CSPRNG Design Architecture

**Figure 6:**
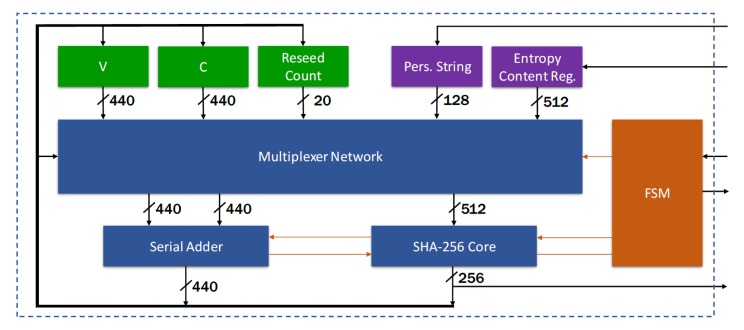
CSPRNG (Hash DRBG) design architecture developed.

The architecture of the proposed CSPRNG IP-core, meaning of the Hash DRBG with optimized SHA-256 core, is shown in Figure 6. The proposed design is based on the following building blocks:

- state registers for 440-bits *V*, 440-bits *C* and 20-bits *reseed counter*; in addition to them, a 128-bits register is available to store optional personalization string (i.e., to randomize the internal state), while a 512-bits entropy register is used to store the input entropy content for a size larger than necessary: to ensure the minimum number of entropy bits per bit string, *instance* requires 394 bits, while *reseed* requires 256 bits;
- a SHA-256 core, with 512-bits input and 256-bits output; the core is designed to execute operations on a 512-bits block of a message in 67 clock cycles; if the message is composed by a single block (*length* ≤ 443 bits), the complete Hash is performed in 67 clock cycles, while if the message is longer, it must be divided in blocks to be processed sequentially;
- a serial adder with 440-bits inputs and modulo 440-bits output; since the adder always runs in parallel with the SHA-256 core, which at least requires 67 cycles to output data, there were no need to implement a low latency adder; intermediate results are stored to one of the input registers to minimize the area occupation;
- multiplexer network to address all data in internal state and from the previous operation to the inputs of the SHA-256 core and adder; as an example, consider the following configuration, where the adder calculates at every Hash cycle the incremented value of *V* and provides this data to the input of the SHA-256 for it to be hashed:
  sha_256_in = adder_out || 1’d1 || 7’d0 || 64’d440
  adder_x = adder_out
  adder_y = 440’d1
- a Finite State Machine (FSM), which controls the flow of operations: it is articulated in three main branches (i.e., *instance*, *reseed* and *generate*), and after the completion of a command, it remains stable in idle mode until another command is issued;
- a DRBG self test module (i.e., not shown in Figure 6), which provides built-in self-test functionalities to diagnose possible failures; multiplexers are used to feed the procedures with known values, and compare logic check if outputs match with the expected values.

The *instance* procedure acquires 512 entropy bit from the entropy content input and then hashes eight blocks to create the internal state. With τ equal to the number of clock cycles necessary to acquire 8 bitt from the entropy content input, total execution time is approximately:

(10) $$t_{instance}=\left(64\cdot \tau +8\cdot 67\right)\cdot T_{clk}=\left(64\cdot \tau +536\right)\cdot T_{clk}$$

The *reseed* procedure acquires 384 entropy bit from the entropy content input and then hashes 8 blocks to update the internal state. Execution time is approximately:

(11) $$t_{reseed}=\left(48\cdot \tau +8\cdot 67\right)\cdot T_{clk}=\left(48\cdot \tau +536\right)\cdot T_{clk}$$

In the *generation* phase, if a personalization string is inserted by the user, a new value of *V* is immediately calculated before generating output bits. This operations requires a sum and two hash cycles. Since the serial adder latency is 14 clock cycles:

(12) $$t_{gen\_pers\_string}=\left(14+2\cdot 67\right)\cdot T_{clk}$$

After generation a new state is derived within the same time:

(13) $$t_{gen\_new\_state}=t_{gen\_pers\_string}=\left(14+2\cdot 67\right)\cdot T_{clk}$$

For a clock frequency of 100 MHz and τ = 1, these values result to be:

$$\begin{array}{cc} \; & t_{instance}=6.000\;\mu s\\ \; & t_{reseed}=5.840\;\mu s\\ \; & t_{gen\_pers\_string}=1.480\;\mu s\\ \; & t_{gen\_new\_state}=1.480\;\mu s \end{array}$$

For the same clock frequency and τ = 8:

$$\begin{array}{cc} \; & t_{instance}=10.480\;\mu s\\ \; & t_{reseed}=9.200\;\mu s\\ \; & t_{gen\_pers\_string}=1.480\;\mu s\\ \; & t_{gen\_new\_state}=1.480\;\mu s \end{array}$$

## 5. Results

In order to validate the CSPRNG IP-core, evaluation of the randmoness degree of the sequences generated was obtained by using the NIST Statistical Test Suite [14]. The test suite ran on a sequence of 128 MB acquired from the CSPRNG with the following strategy:

- every *generation* command requests four blocks, i.e., 1024 bits;
- during a seed lifetime, 1000 *generation* commands are issued;
- the *reseed* operation is commanded 1000 times to reseed the generator.

In this way the total number of acquired bits is $n_{bit}=1024\cdot 1000\cdot 1000=$ 1,024,000,000. This sequence has been then converted to a binary file, which is subsequently given as input to the test suite. The NIST Statistical Test Suite parameters and the corresponding results (using α=0.01) on the 128 MB data block are collected on Table 7.

Three technologies were identified as potential targets for characterization of the CSPRNG hardware accelerator IP-core, one FPGA and two ASIC standard-cell: Intel Stratix IV FPGA, 45 nm Silvaco [15], and 7 nm Artisan TSMC [16]. In all of these cases different implementation effort corners were tested, in order to evaluate the trade-off between throughput and area. The synthesis performed on Intel Stratix IV (EP4SGX230KF40C2) FPGA technology with high performance constraints, configuring a single instance of the SHA-256 core, provides a maximum operating frequency of 180 MHz; a throughput of 690 Mbps; and an overall resource utilization of 4713 ALMs. The 45 nm Silvaco ASIC standard-cell implementation increases its throughput to 3.82 Gbps, since the maximum frequency is 1 GHz (being the critical path in the SHA-2 sub-block), with a logical complexity of 49.19 kGE. Finally, the proposed design, with a single SHA-256 core, brought on the 7 nm Artisan ASIC standard-cell reaches a throughput value of 19.67 Gbps, given a maximum clock frequency of 5.15 GHz, requiring an overall complexity of 46.56 kGE.

**Figure 7:**
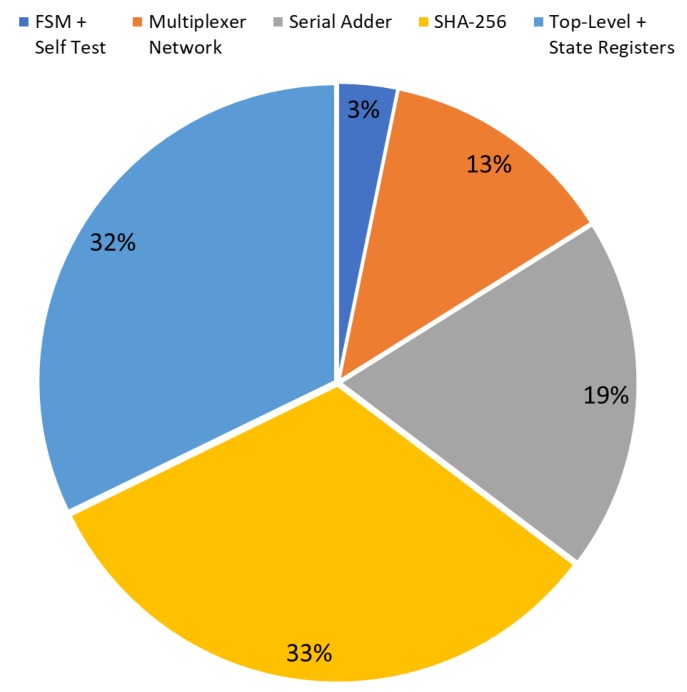
CSPRNG (Hash DRBG) IP-core occupation diagram on 45 nm ASIC technology (kGE based).

**Figure 8:**
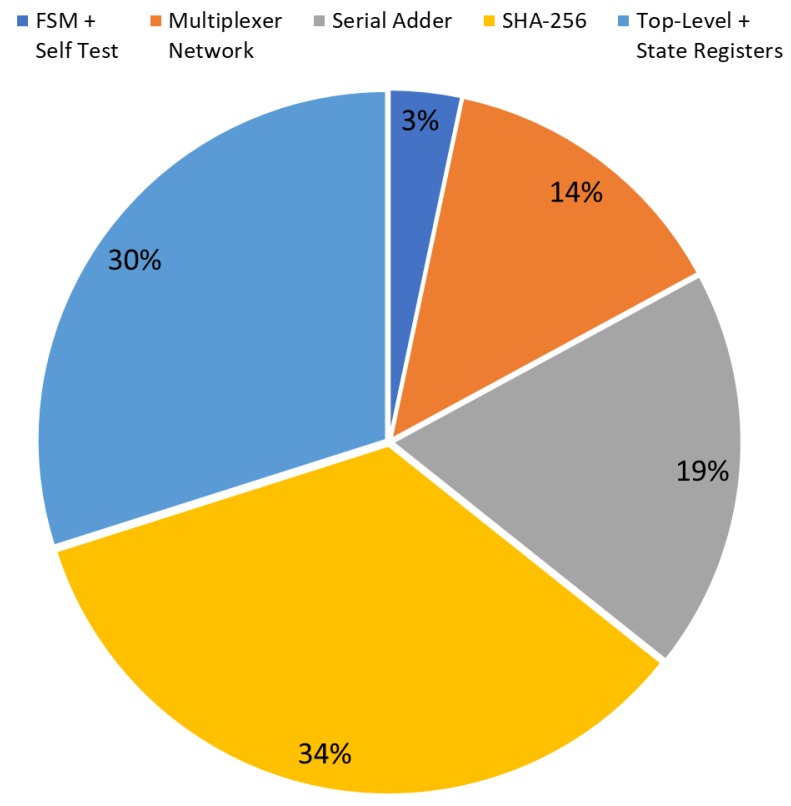
CSPRNG (Hash DRBG) IP-core occupation diagram on 7 nm ASIC technology (kGE based).

The diagrams in Figure 7 and Figure 8 show the occupation percentage for the different parts of the architecture proposed respectively for 45nm and 7nm ASIC target technologies.

## 6. Conclusions

This paper presented the architecture and implementation of a high performance digital Cryptographically Secure Pseudo-Random Number Generator (CSPRNG). Specifically, a Hash-based Deterministic Random Bit Generator (DRBG) circuit was presented, following recommendation given by NIST in [6], using SHA256 cryptographic primitive. CSPRNG is a key component to implement efficient cybersecurity applications for authentication, confidentiality and message integrity. In addition, the security of critical information exchanged between the different subsystems in modern vehicles is proving to be a key issue in the automotive sector: more advanced and complex devices and sensors provide the platform for active assistance and security on which passengers rely, so it is crucial to ensure that these systems are adequately protected from cyber attacks. Hash algorithm selection was done according to a trade-off analysis on throughput, area and security strength: among the solutions able to satisfy the security requirements, the SHA-256 core was proved to be the most efficient solution in terms of throughput-complexity ratio. The detailed description of the optimized SHA-256 core architecture being developed for DRBG circuit implementation is also given. The proposed CSPRNG IP-core was tested by means of NIST Statistical Test Suite, thus stating that the sequences of bits generated cannot be distinguished from a true random sequence of numbers, and therefore validating its use for cryptographic applications. It has been also implemented on FPGA and ASIC standard-cell technologies for characterization: on Intel Stratix IV FPGA it is reported a throughput of 690 Mbps at 180 MHz with a maximum occupation of 4713 ALMs, on 45 nm ASIC standard-cell [15] the throughput is equal to 3.82 Gbps at 1 GHz with a logic complexity of 49.19 kGE, and finally on 7 nm ASIC standard-cell [16] the throughput reaches a value of 19.67 Gbps at 5.15 GHz with the logic complexity of 46.56 kGE.

## Figures and Tables

**Table 1:** Hash DRBG mechanisms parameters (SHA2 only).

	SHA2 Algorithm			
	SHA-224	SHA-256	SHA-384	SHA-512
Highest Security Strength	192 bits	256 bits	256 bits	256 bits
Output Block Length (outlen)	224 bits	256 bits	384 bits	512 bits
Min. Entropy for instance and reseed	192 bits	256 bits	256 bits	256 bits
Seed Length (seedlen)	440 bits	440 bits	888 bits	888 bits
Max. Num. of Bit per Request	$2^{19}$	$2^{19}$	$2^{19}$	$2^{19}$
Max. Num. of Request between reseeds	$2^{48}$	$2^{48}$	$2^{48}$	$2^{48}$

**Table 2:** CTR DRBG mechanisms parameters. $B=(2^{ctrl\_len}-4)$blocklen.

	AES Algorithm			
	3 Key TDEA	AES-128	AES-192	AES-256
Highest Security Strength	112 bits	128 bits	192 bits	256 bits
Input/Output Block Length (blocklen)	64 bits	128 bits	128 bits	128 bits
Key Length Length (keylen)	168 bits	128 bits	192 bits	256 bits
Counter Field Length (ctr_len)	4 <ctr_len <blocklen			
Min. Enctropy for instance and reseed	112 bits	128 bits	192 bits	256 bits
Seed Length (seedlen)	232 bits	256 bits	320 bits	384 bits
Max. Num. of Bit per Request	min(*B*, $2^{13}$)	min(*B*, $2^{19}$)	min(*B*, $2^{19}$)	min(*B*, $2^{19}$)
Max. Num. of Request between reseeds	$2^{48}$	$2^{48}$	$2^{48}$	$2^{48}$

**Table 3:** SHA2 IP-core specifications.

SHA2 Algorithm	Area	Latency per Block	Output Block Size
SHA-224	15 kGE	67 clock cycles	224 bits
SHA-256	15 kGE	67 clock cycles	256 bits
SHA-384	30 kGE	83 clock cycles	384 bits
SHA-512	30 kGE	83 clock cycles	512 bits

**Table 4:** AES IP-core specifications.

AES Algorithm	Area	Latency per Block	Output Block Size
AES-128	11 kGE	11 clock cycles	128 bits
AES-256	12.5 kGE	15 clock cycles	128 bits

**Table 5:** Canonical and Optimized SHA-256 implementation results.

Canonical	Area	Max. Frequency	Optimized	Area	Max. Frequency
45 nm ASIC	16.85 kGE	640 MHz	45 nm ASIC	15.38 kGE	1.00 GHz
7 nm ASIC	17.18 kGE	3.15 GHz	7 nm ASIC	15.45 kGE	5.15 GHz

**Table 6:** SHA-256 sub-block implementation results.

Sub-Block	$T_{4\cdot \mathrm{CSA}}$	$T_{2\cdot \mathrm{CSA}+1\cdot \mathrm{CLA}}$	$T_{2\cdot \mathrm{CLA}}$	$T_{5\cdot \mathrm{CLA}}$
Max. Latency	606.06 ps	833.33 ps	714.29 ps	1111.11 ps

**Table 7:** NIST Statistical Test Suite parameters and results.

Test	Block/Template Length	Pass Rate
Frequency (Monobit)	-	0.9924
Frequency Within a Block	256	0.9876
Runs	-	0.9901
Longest-Run-of-Ones in a Block	-	0.9878
Binary Matrix Rank	-	0.9901
Discrete Fourier Transform (Spectral)	-	0.9874
Non-overlapping Template Matching	10	[0.9801–0.9974]
Overlapping Template Matching	10	0.9848
Maurer’s Universal Statistical	-	0.9901
Linear Complexity	1024	0.9900
Serial	16	0.9825, 0.9876
Approximate Entropy	10	0.9901
Cumulative Sums (Cusums)	-	0.9901
Random Excursions	-	[0.9826–0.9947]
Random Excursions Variant	-	[0.9875–0.9975]
